# Kidney Allograft Dysfunction Due to John Cunningham (JC) Virus Nephropathy

**DOI:** 10.7759/cureus.32021

**Published:** 2022-11-29

**Authors:** Neeraj Sharma, Samer Abdulkhalek

**Affiliations:** 1 Department of Nephrology, University of Southern California, Los Angeles, USA; 2 Department of Nephrology, University of Southern California Keck School of Medicine, Los Angeles, USA

**Keywords:** polyomavirus-associated nephropathy, nephropathy, jc polyomavirus, immunosuppression, renal allograft, viral infection, kidney transplantation, transplantation

## Abstract

John Cunningham (JC) polyomavirus-associated nephropathy (JC-PVAN) is a rare cause of polyomavirus-associated nephropathy (PVAN). Although BK polyomavirus (BKPyV) is a relatively proven common infection post kidney transplantation, JC polyomavirus (JCPyV) infection and its impact on graft function have been less studied. Here, we report a case of a deceased donor kidney transplant recipient who was diagnosed with allograft dysfunction due to JC-PVAN six years after transplantation. JC viremia resolved after a reduction in immunosuppression and treatment with intravenous immunoglobulin (IVIG); however, she developed an acute cellular rejection with moderate fibrosis resulting in chronic kidney disease in the allograft.

## Introduction

Polyomavirus-associated nephropathy (PVAN) is an important cause of graft dysfunction and graft loss [1]. The definitive diagnosis of PVAN requires an allograft biopsy, which shows intranuclear viral inclusions within tubular epithelial cells and positive immunohistochemical staining for viral antigens [2]. The majority of PVAN after kidney transplantation is due to the BK virus [3]. However, another type of polyomavirus, John Cunningham (JC) polyomavirus, which establishes latency in the kidney, is also associated with PVAN. Despite JC virus seropositivity being ubiquitous in the general population, it remains a rare cause of polyomavirus nephropathy in kidney transplant recipients with an incidence of <1% [4]. Intense immunosuppression is considered the most important risk factor. However, patient determinants (older age, male gender, seronegative recipient) and viral factors (genotype, serotype) may have a contributory role [5]. Our case demonstrates that JC polyomavirus-associated nephropathy (JC-PVAN) can eventually lead to chronic kidney disease in the allograft as a result of fibrosis and tubular atrophy, despite aggressive treatment.

## Case presentation

A 54-year-old female with end-stage kidney disease due to autosomal dominant polycystic kidney disease underwent a deceased donor kidney transplant. Prior to the transplant, the patient had been on peritoneal dialysis for three years. Human leukocyte antigen (HLA) mismatch was A2, B2, and DR1. Induction therapy consisted of anti-thymocyte globulin 4 mg/kg over three days and methylprednisolone, followed by maintenance therapy with tacrolimus, mycophenolate mofetil, and prednisone. The patient experienced immediate graft function. Cytomegalovirus (CMV) status was positive for both donor and recipient and the patient completed a six-month course of valganciclovir for CMV prophylaxis.

**Figure 1:**
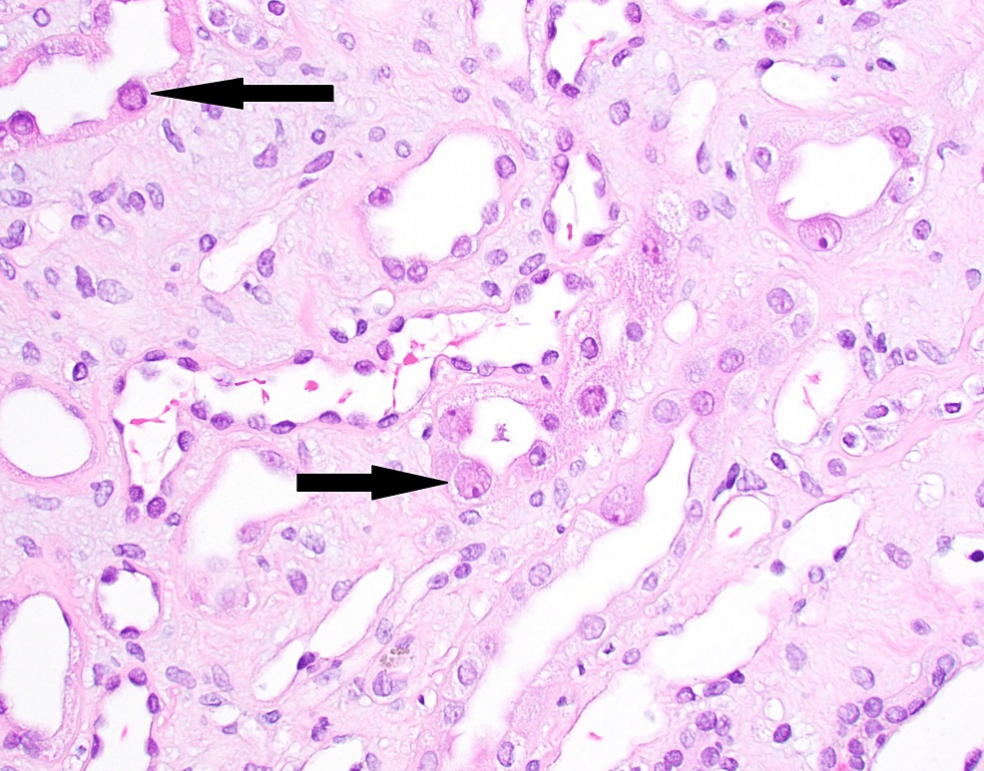
Medullary tubules with viral cytopathic changes (hematoxylin and eosin stain, 400x magnification). Black arrows indicate medullary tubules with viral cytopathic changes.

**Figure 2:**
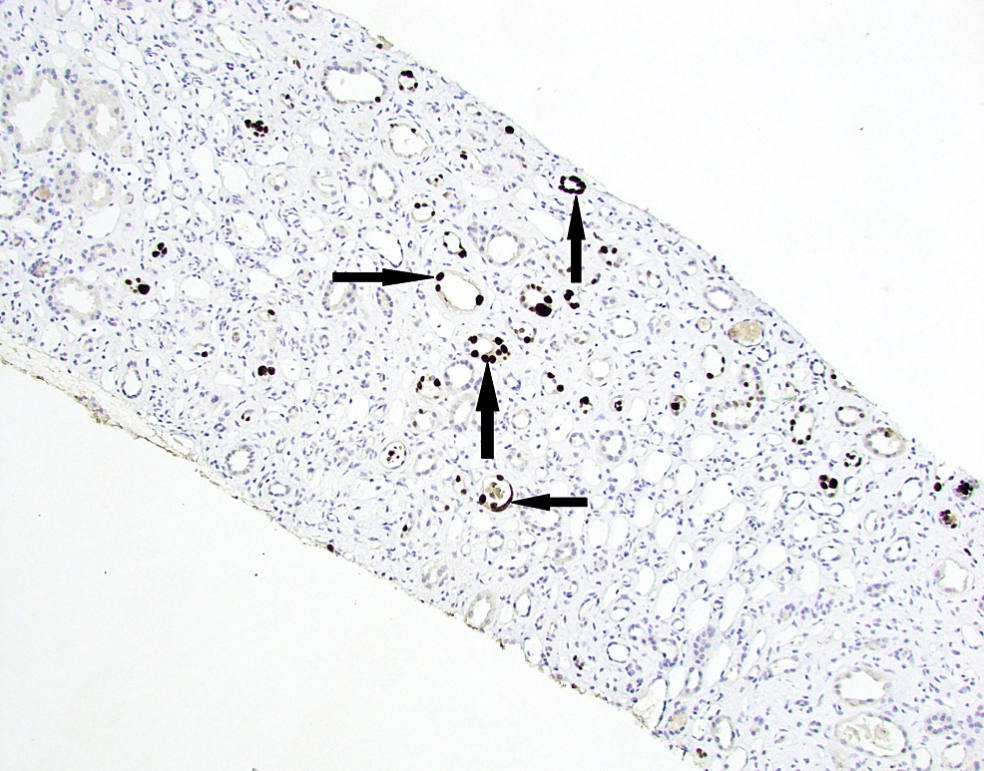
Multifocal nuclear staining (SV40 antigen by immunohistochemistry, 100x magnification). Black arrows denote positive SV40 staining in medullary tubules.

Approximately six years after the transplant, the patient presented with acute kidney injury with a creatinine of 3.3 mg/dl compared to a baseline creatinine of 1.5 mg/dl. During this time, the patient was receiving tacrolimus 6 mg twice daily, mycophenolic acid 360 mg twice daily, and prednisone 5 mg daily. Workup for allograft dysfunction revealed negative quantitative urine BK polymerase chain reaction (PCR) and negative plasma CMV PCR testing. Donor-specific antibodies (DSAs) were not found. Urinalysis was negative for protein and red blood cells. Urine cytology was negative for decoy cells. A transplant kidney ultrasound did not reveal hydronephrosis, transplant renal artery stenosis, or ureteral stenosis. Tacrolimus levels over the past three months were 5-8 ng/ml. In view of persistent allograft dysfunction despite a negative workup, a transplant kidney biopsy was performed. Biopsy revealed borderline acute T cell-mediated rejection (t1, i2, v0), acute focal interstitial inflammation, and scattered medullary tubules with viral cytopathic changes (Figure 1). Immunohistochemistry staining with SV40 reagent was positive in many cells (Figure 2). JC virus DNA by quantitative real-time PCR in the blood was tested and returned positive with 8,524 copies/mL. JC virus quantitative PCR in urine was detected at greater than 35,000,000 copies/mL. The diagnosis of JC nephropathy was made. Immunosuppression was adjusted, which included discontinuation of mycophenolic acid and reduction of the tacrolimus target levels to between 3 and 5 ng/ml (one-half of the original level). In addition, the patient was initiated on intravenous immunoglobulin (IVIG) 500 mg/kg weekly for four weeks.

The patient also underwent a lumbar puncture and brain MRI to evaluate for CNS involvement of the JC virus. There were no specific concerning findings on the MRI of the brain and the lumbar puncture was negative for any infectious process. Repeat JC virus in urine was still greater than >35,000,000 copies/mL. However, one month after diagnosis, blood JC virus PCR had decreased to 3,924 copies/ml. About three months after diagnosis, JC virus blood further decreased to 2,500 copies/mL. Approximately nine months after the initial biopsy, the JC virus PCR in the blood had decreased to 1,674 copies/mL. Initially, our patient was treated with IVIG 500 mg/kg weekly infusions for the first four weeks, which was tapered to every two weeks for the next eight weeks, then once every month. With treatment, the allograft function did not return to baseline; however, it stabilized at a serum creatinine of 3.0 mg/dL. Therefore, a second transplant kidney biopsy was performed approximately one year after the initial biopsy, which revealed a Banff 1A acute T cell-mediated rejection (i2, t2, Vo). Immunohistochemistry staining for SV40 staining was negative. In addition, the biopsy was also notable for marked interstitial fibrosis and tubular atrophy. The patient was treated with pulse methylprednisolone over three days. Repeat single antigen testing revealed DSAs to DR51 at 1,542 MFI and DQ4 at 7,844 MFI. JC virus PCR in blood at the time of the second biopsy was <500 copies/mL. However, the urine JC virus PCR remained positive. IVIG was discontinued and the patient was maintained on tacrolimus with goal targets of 3-5 ng/mL, azathioprine 50 mg daily, and prednisone 5 mg daily. Despite the significant interstitial fibrosis that had developed, our patient’s allograft function remained stable with an estimated glomerular filtration rate (eGFR) between 25 and 30 ml/m2.

## Discussion

JC-PVAN is a rare entity seen in less than 1% of transplanted kidney biopsies, even though JC viruria is more common than BK viruria in healthy non-transplant adults. However, in the transplant population, PVAN is mainly caused by the BK virus. Therefore, JC-PVAN has been recognized as the cause in less than 3% of all reported cases [6-8]. As a result, there are limited reports of JC virus nephropathy occurring in kidney transplant recipients. To date, the largest case series on JC-PVAN by Wiegley et al. reported seven kidney transplant patients with biopsy-proven JC-PVAN [9], which demonstrates a few similarities to the previous study done by Drachenberg et al. [4]. Similarities included the following: (a) JC-PVAN presenting much later than BK-PVAN; (b) some JC-PVAN patients did not have viremia at the time of biopsy; (c) some patients were treated for acute rejection prior to the development of JC-PVAN; (d) both studies found that the predominant histologic pattern was chronic inflammation within areas of fibrosis. The study by Wiegley et al. identified one case of JC-PVAN, which mimicked acute T-cell rejection. In addition, Wiegley et al. also noted that their patients had significantly higher serum JC viral loads than Drachenberg et al. (average of 87,000 versus 2,000 copies/mL). Our case reveals similarities to the patients presented by Wiegley et al., such as acute allograft dysfunction, late presentation post-transplant, and a high serum JC viral load.

Given that nearly 60% of adults are seropositive for the JC virus [10], it is likely that acute infection is due to latent asymptomatic infection, especially since re-activation of the latent virus is critical in the development of JC polyomavirus nephropathy. A large case series by Lopez et al. [11] that included 186 transplant patients showed that although the incidence of BK virus and JC virus viruria was similar, the JC virus serotype did not cause viremia or polyomavirus nephropathy, unlike with BK virus.

The most dreadful manifestation of JC viremia is progressive multifocal leukoencephalopathy (PML). Our patient was screened and ruled out for PML.

Diagnosis of JC-PVAN requires both histological and serological evidence of the JC virus. The biopsy findings in JC-PVAN tend to be indistinguishable from BK-PVAN. As a result, kidney biopsy has many inherent limitations, as described by Yang et al. [5]. For example, because of the focal disease pattern, there is a high risk of sampling non-infected tissues resulting in a false negative biopsy finding. Furthermore, in a more advanced pattern of injury, viral inclusions can be lacking, which further obscures an accurate diagnosis. Immunohistochemistry staining in the biopsy relies on a cross-reactive antibody against SV40, an epitope located in the regulatory viral proteins, which is common to the BK virus, JC virus, and SV40. Therefore, the diagnosis of JC-PVAN is validated when a histological analysis is combined with quantification testing of the JC virus in the serum and urine to confirm the infection. However, it is important to understand that single urine or serum testing is of limited value, and rather a trend of viruria or viremia aids in identifying patients at the highest risk of infection. It is important to determine viruria or viremia at the time of biopsy since the histology of PVAN can resemble acute cellular rejection, which can inherently result in the wrong treatment.

In terms of treatment, there is no approved treatment or prospective randomized trials evaluating the efficacy of potential therapies for JC-PVAN. The mainstay of treatment remains the reduction of immunosuppressive therapy [12]. The treatment for the patient presented in this case included discontinuation of mycophenolate, reduction of the tacrolimus trough levels, and addition of IVIG. Despite aggressive treatment, the allograft function did not improve; however, it stabilized to eGFR 25-30 ml/m2. Of all studies searched, none has shown any improvement in allograft function after the treatment of JC-PVAN. Reduction in immunosuppression has risks, as in our patient who developed a Banff 1A acute cellular rejection. A systematic review by Johnston et al. [13] showed that the rate of acute rejection following a reduction in immunosuppression after diagnosis of BK-PVAN was between 6% and 75%. Fortunately, the rejection episodes were treated without recurrence of PVAN such as in our patient. Another treatment strategy would be the conversion of tacrolimus to sirolimus for persistent viruria.

## Conclusions

Given that JC polyomavirus typically presents many years after transplantation, we suggest having a high index of suspicion for JC infection in patients presenting with allograft dysfunction and hematuria. Furthermore, due to the development of interstitial fibrosis and tubular atrophy in patients with JC-PVAN, it may be prudent to ensure patients' immunosuppression is reduced, if possible, to prevent this rare but clinically significant infection.
